# Cost-Effectiveness Analysis of Seven Measures to Reduce Tail Biting Lesions in Fattening Pigs

**DOI:** 10.3389/fvets.2021.682330

**Published:** 2021-09-07

**Authors:** Jarkko K. Niemi, Sandra A. Edwards, Dimitris K. Papanastasiou, Deborah Piette, Anna H. Stygar, Anna Wallenbeck, Anna Valros

**Affiliations:** ^1^Bioeconomy and Environment Unit, Natural Resources Institute Finland (Luke), Seinäjoki, Finland; ^2^School of Natural and Environmental Sciences, Newcastle University, Newcastle upon Tyne, United Kingdom; ^3^Department of Environmental Sciences, University of Thessaly, Larissa, Greece; ^4^Department of Biosystems, KU Leuven, Leuven, Belgium; ^5^Bioeconomy and Environment Unit, Natural Resources Institute Finland (Luke), Helsinki, Finland; ^6^Department of Animal Environment and Health, Swedish University of Agricultural Sciences, Uppsala, Sweden; ^7^Research Centre for Animal Welfare, University of Helsinki, Helsinki, Finland

**Keywords:** swine, tail biting, economic losses, simulation, prevention, costs, benefits

## Abstract

Tail biting is an important animal welfare issue in the pig sector. Studies have identified various risk factors which can lead to biting incidents and proposed mitigation measures. This study focused on the following seven key measures which have been identified to affect the risk of tail biting lesions: improvements in straw provision, housing ventilation, genetics, stocking density, herd health, provision of point-source enrichment objects, and adoption of early warning systems. The aim of this study was to examine whether these selected measures to reduce the risk of tail biting lesions in pig fattening are cost-effective. The problem was analyzed by first summarizing the most prospective interventions, their costs and expected impacts on the prevalence of tail biting lesions, second, by using a stochastic bio-economic model to simulate the financial return per pig space unit and per pig at different levels of prevalence of tail biting lesions, and third by looking at how large a reduction in tail biting lesions would be needed at different levels of initial prevalence of lesions to cover the costs of interventions. Tail biting lesions of a severity which would require an action (medication, hospitalization of the pig or other care, or taking preventive measures) by the pig producer were considered in the model. The results provide guidance on the expected benefits and costs of the studied interventions. According to the results, if the average prevalence of tail biting lesions is at a level of 10%, the costs of this damaging behavior can be as high as €2.3 per slaughtered pig (~1.6% of carcass value). Measures which were considered the least expensive to apply, such as provision of point-source enrichment objects, or provided wider production benefits, such as improvements in ventilation and herd health, became profitable at a lower level of efficacy than measures which were considered the most expensive to apply (e.g., straw provision, increased space allowance, automated early warning systems). Measures which were considered most efficient in reducing the risk of tail biting lesions, such as straw provision, can be cost-effective in preventing tail biting, especially when the risk of tail biting is high. At lower risk levels, the provision of point-source objects and other less costly but relatively effective measures can play an important role. However, selection of measures appropriate to the individual farm problem is essential. For instance, if poor health or barren pens are causing the elevated risk of tail biting lesions, then improving health management or enriching the pens may resolve the tail biting problem cost-effectively.

## Introduction

Tail biting is an important multifactorial animal welfare issue in the pig sector. Various risk factors can lead to biting incidents and therefore multiple measures may be used to control the problem. Farm management practices and housing conditions, such as inadequate access to enrichment, slatted floors, high stocking density, inadequacies in the ventilation, water or feed supply, mixing of animals, not removing the biter causing the problem, and genetic background of the pigs used are known to affect the risk of tail biting [e.g., (1–3)].

The magnitude of the problem varies across countries, cases and classification criteria [e.g., (2, 4–6)]. Observational studies have reported fresh tail damage in 11%, and a severe lesion in 1.3%, of pigs at a Finnish slaughter line (7); some injury or shortening of the tail in up to 7.2%, and a severe lesion in up to 1.9%, of pigs at a Swedish slaughterhouse (8); and a lesion in 25.4%, and a severe lesion or necrosis in 1.6%, of pigs at a German abattoir (6). The most extreme report is from an Irish study (9) in which as many as 72.5% of pigs examined in a slaughterhouse had a detectable tail lesion, although only 2.5% of pigs showed a severe tail lesion. Meat inspection records have reported tail lesions for example in 0.5–1.0% of Danish (10, 11) and 4% of Norwegian pigs (12). These differences in the prevalence of tail biting might be due to different evaluation criteria. Official meat inspection records typically show much lower prevalence than more detailed observations from slaughter lines or farms [see e.g., (6, 8, 13)]. Inspection records often contain only cases which can compromise meat hygiene, whereas data collected by scientists for a specific purpose can be more detailed and may include also less severe cases.

Previous studies have reported that tail biting lesions cost the UK pig industry £3.51 million per year [information cited by Ref. (14)]; about €2,400 per year for a typical finishing herd in the Netherlands, (<1% of the sale value of the pigs, assuming 2% of pigs suffer from the lesions) (15); and €18.96 per victim of tail biting in Danish conditions (~15% of the sale value of a pig with lesions) (16). Recently a €1.1 reduction of the mean annual farm profit per produced pig (−15.1%) in Irish farms with a high prevalence of severe tail lesions was reported (17). An unpublished literature review by the authors suggests that the costs of tail biting lesions across Europe are around €2.0 (±€1.4) per finished pig (1–3% of the sale value of a pig).

Despite the importance of the problem, there is little evidence on the cost-effectiveness of interventions to reduce the risk of tail biting lesions in pigs. This paper contributes to the literature by analyzing financial aspects of interventions to control tail biting in fattening pigs. Financial analysis can provide useful information for decision makers who are considering whether to adopt animal welfare improving technologies and practices. Circumstances which can lead to adoption of improved management practices include, for example, productivity gains obtained as a consequence of the implemented technology or practice, or a price premium which provides an additional incentive to the pig producer to upgrade animal welfare. However, investments in animal welfare can be financially prohibitive if extra revenues and cost savings obtained from applying an intervention are smaller than the additional costs incurred (18). Although tail biting decreases the performance of pigs and causes extra costs [e.g., (19)], it may be tempting for a producer to tolerate the problem or adopt suboptimal measures because it can be even more costly to eliminate the root cause. This may be the case, for instance, when tail docking is applied as a preventive tool without removing the root causes of the tail biting problem.

The aim of this study was to examine whether selected measures to reduce the risk of tail biting lesions in pig fattening are cost-effective. The problem was analyzed by first reviewing and describing the rationale of the most prospective interventions, their costs and expected impacts on the incidence of tail biting; second, by using a stochastic bio-economic model to simulate the return on pig space unit at different levels of occurrence of tail biting lesions which would require an action (veterinary treatment, preventive intervention, or other specific care) from the pig producer; and third, by looking at how large a reduction in tail biting lesions would be needed at different levels of prevalence of lesions to cover the costs of interventions.

## Materials and Methods

### Computational Model to Assess the Costs of Tail Biting

#### Objective Function

The costs of tail biting lesions were assessed by using a dynamic simulation model. The benefits of interventions were the expected savings due to reducing tail biting lesions. The model maximized the return on pig space by optimizing the timing of slaughter under a predefined risk of tail biting. The stochastic model simulated whether an individual pig in the pen becomes a victim of tail biting (see section Biological Model for a Tail Biting Outbreak in the Pen). Only lesions which are severe enough to require a pig producer's intervention, such as medication or other veterinary care, hospitalizing the pig, taking preventive actions or some other form of intervention, were considered in the model. The model also simulated the weights of pigs on a daily basis by taking into account whether the pig has been bitten and the current weight of the pig, as well as a model component which simulates economic and biological parameters associated with tail biting, including parameters such as mortality rates, other health disorders, carcass condemnations, carcass value, feed and other costs and revenues associated with fattening of both bitten and non-bitten pigs. The model was run under several scenarios, as presented in section Simulations. Because the model is stochastic, the exact tail biting status and input-output ratio was known for each pig only after slaughtering it. The modeling approach was able to take into account uncertainty related to the occurrence of tail biting lesions.

The model was designed to represent a fattening pig compartment where all piglets arrive on the same day and weigh on average 25 kg at arrival. The pigs are reared until reaching a slaughter weight which is resolved by the model and, after a cleaning break, a new batch of piglets is brought in. In the model, the expected net present value of the current pig rearing facility (value per pig space unit) is maximized. The value is the discounted net present value of cash flows from selling pigs at slaughter weight minus the price of purchased weaned piglets, the feed costs and costs associated with the control and treatment of events of tail biting in the pen at a given planning horizon *t* = 1, …, *T*. The costs of preventive measures, however, were considered outside the model by using information presented in section Characterization of Interventions Studied.

This modeling approach was used because the net present value is considered as the best criterion for ranking investments or projects. Because the model maximizes the productive value of an asset (the pig farm) by using cash flows, the focus is on return on investment, and it is not necessary to specify fixed assets. Because of this approach, changes in productivity because of tail biting lesions are reflected in the financial results and the model also allows us to review the value of information under rapid detection methods [see (20) for discussion]. A similar approach has been used previously to analyse pig herd management (21).

More formally, the economic model follows the equation:

(1) $$\begin{array}{r} V_{1}\left({\text{x}}_{1}\right)=E\left(\underset{{\text{u}}_{t}\left({\text{x}}_{t}\right)}{\max}\left\{R_{t}\left({\text{x}}_{t},{\text{u}}_{t}\right)+\beta V_{t+1}\left({\text{x}}_{t+1}\right)\right\}\right)\;for\;t=1,\ldots ,T \end{array}$$

subject to: **x**_*t*+1_ =*g*(**x**_*t*_**, u**_*t*_, ε) (transition equations)

**x**_1_ and *V*_*T*__+1_(**x**_T+1_) given (initial state and the terminal value given)

where *V*_1_(*x*_1_) is the value of pig space as a function of the current state vector ${\text{x}}_{t}=\{x_{t,j,w},x_{t,j,TB}\}$ for all *j*; the subscript *t* is the time index, the time unit being 1 day; *x*_*t,j,w*_ is the live weight of the pig; *x*_*t,j,TB*_ is the tail biting status of an animal {tail lesions, no tail lesions}; **u**_*t*_ = {*u*_*t,cull*_, *u*_*t,TB*_} is the control policy; refers to the timing of slaughtering the pig and to the rule to manage the risk of tail biting; *R*_*t*_(.) is the one-period return function; β is the discount factor; *E*(.) is the expectations operator; *V*_*t*+__1_(**x**_t+1_) is the next-period value function; *g*(.) represents the pig growth model, the slaughtering decision and transition equation for tail biting; the “error” term ε refers to the variation in the pigs' carcass composition and growth; *V*_*T*__+1_(**x**_*T*+1_) is the value of the pig space unit after the terminal period *T*, and **x**_1_ is the state at the beginning of the planning horizon (set at a 25 kg piglet). The optimal management pattern is defined as a function of state variables.

The model was structured so that it simulates many batches instead of just one batch and until the model has converged. The model was normalized per pig space unit and solved using a policy iteration method. The solution procedure utilizes first-order conditions for model convergence [i.e., *V*_*t*_(**x**_**t**_) = *V*_*t*__+1_(**x**_**t+1**_)]. In order to solve the optimisation problem, there must be a discount factor <1. Model results indicate the productive value of a pig space unit over an infinite time horizon. After running the model, this value was converted into € per pig or € per pig space unit per year, by running the model so that the contribution of one pig to the value function (Equation 1) was obtained.

A number of parameters were specified for the model (Table 1). Pig growth was modeled using a modified version of the previously developed model (24). The impact of tail biting on pig growth is divided into typical and severe impacts according to data from a progeny test station (19, 25, 26). Market value of a carcass is based on a scheme, which pays a premium or discount per kg of meat according to the carcass weight and red meat percentage. However, condemned parts of a carcass are not paid for. A condemnation may also result in an additional price discount.

**Table 1:** Parameter values used in the model to simulate the economic consequences of tail biting in a pen.

**Parameter**	**Value**
Typical duration of treatment per bitten pig^a^	5 days
Estimated duration of illness in a bitten pig^a^	7 days
Share of bitten pigs moved to a hospital pen and kept there until slaughter^a^	15%
Reduction in the daily weight gain after being bitten (19, 22)	11%
Percentage of bitten pigs dead or disposed (15)	2.1%
Percentage of a bitten pig's meat mass lost due to carcass condemnations (7)	0.7%
Annual discount rate β	0.94
Materials, medicine and veterinary fees, € per bitten pig updated from Ref. (16)	6.00
Extra labor and other cost due to a hospital pen, € per bitten pig updated from Ref. (16)	7.61
Average cost related to premature disposal or death, € per pig^b^	15.00
Cost of labor, € per hour (23)	16.00
Price of pigmeat, € per kg^c^	1.63
Price of a piglet, € per animal (23)	55.00
Average price of feed, € per kg (23)^d^	0.25

a *Based on consultation with veterinarians and on health care data from pig farms*.

b *Raatonetti www.raatonetti.fi*.

c *Historical annual market prices for pig carcass grade S in the E.U. in euro/100 kg carcass. https://ec.europa.eu/info/sites/info/files/food-farming-fisheries/farming/documents/historical-pig-prices-eu_en.pdf*.

d *Feed composition in the growth model was based on Finnish feeding recommendations (https://portal.mtt.fi/portal/page/portal/Rehutaulukot/feed_tables_english)*.

While the starting weight of piglets was set on average at 25 kg, the weight at slaughter was solved by the model. In the baseline simulation, the median carcass weight was 88.4 kg and 90% of pigs were between 78.0 and 92.5 kg carcass weight. The median rearing time of batches (i.e., the number of days from the onset until the last pigs in the batch were sent to slaughter) was 122 days in the baseline simulation. Because the model is normalized per pig space and because it is a simulation model, several factors relevant in biological experiments are not presented explicitly, even though they are implicitly reflected in the parameter values. The basic biological information used in the model was obtained as such from the previous study (24). The growth parameters used in the model were originally defined by using data from studies with cross-bred pigs of Large White, Duroc and Yorkshire, and adapted to a generic level of productivity in the current analysis. Feed intake was defined according to the Finnish feeding recommendations and increased from about 1.5 kg/pig/d at 25 kg liveweight up to about 3 kg/pig/d or more around the slaughter weight.

To account for stochasticity, the current simulations were set so that the population-level distribution was represented by 27,000 pigs. Variation between batches was represented by 1,000 batches and variation within batch was represented by a distribution which had 27 observation points. The parameters were based on housing with partly slatted flooring, mechanical ventilation and a batch intake system.

#### Biological Model for a Tail Biting Outbreak in the Pen

To characterize the number of tail bitten pigs in the pen, the probability of one or more biting incidents to occur in the pen was first simulated. Thereafter, the interval between successive biting incidents in the pen was simulated. The occurrence of tail biting in the pen was simulated with a Monte Carlo procedure as a dynamic process, such that the probability of tail biting to occur in the pen depends on how many cases have been observed by the current moment. The probability of at least one more incident to occur in the pen is:

(2) $$\text{Pr}(n_{t,TB})=\{\begin{array}{ll} \alpha_{1} & \text{if }n_{t,TB}\equiv 0\\ \alpha_{2}+\alpha_{3}\ln(n_{t,TB}+1) & \text{if }n_{t,TB}\geq 1 \end{array},$$

where parameters are as defined in Table 2, and parameter values were defined by using data from an earlier study (19, 25, 26). The likelihood of, and the time between, successive tail biting observations was derived by using health records of individual pigs included in the data. The most important items of information were the time and reason of each disease or tail lesion observation for individual pigs.

**Table 2:** Parameters used in the model to simulate the occurrence of tail biting in a pen.

**Parameter**	**Description**	**Values**
*t*	Time index	[0,…, *T*]
*j*	Index identifying an individual animal in the pen	[1, …, *n*]
*n*_t, TB_	Number of bitten pigs in the pen since the arrival of pigs into the pen until current moment *t*	[1, …, *n*]
Pr*_*j*_*(*n_*t, TB*_*)	Probability that there will be *n_*t*_*+1 tail biting incident in the pen	[0, …, 1]
α_1_	Parameter of probability function	Varies by scenario^a^
α_2_	Parameter of probability function	0.54
α_3_	Parameter of probability function	0.11

a *Parameter α_1_ was scaled up or down to achieve the prevalence of tail biting lesions desired for each analyzed scenario*.

**Figure 1:**
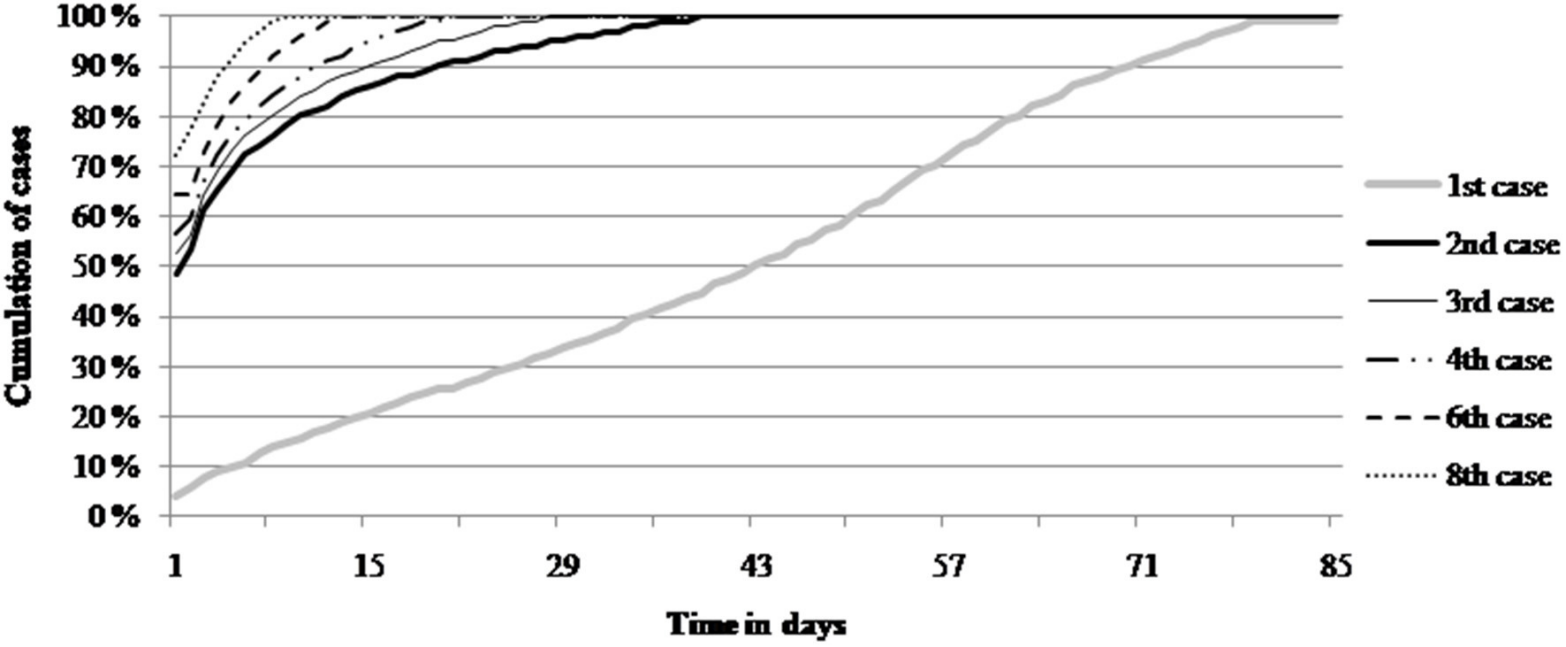
Percentage of the *n*th case of tail biting lesions to have occurred by a certain number of days after observing the *n*−1th case in the pen, and for the first case the number days from the arrival of the pigs into the pen until the occurrence of the first case in the pen [Source: Ref. (25) with data described by Ref. (26)].

The impact of different risk factors for tail biting which are assumed to be present at the farm was modeled by adjusting the parameter, which parametrises the prevalence of tail biting lesions in the optimisation problem presented in Equation 1. Another key element of the model is that tail biting can emerge over time. Our data suggest that tail biting incidents are agglomerated in time, and that the time between successive cases of tail biting in a pen is shorter the more cases that have been observed. According to our data, obtained from Finnish (and Swedish) progeny test stations with undocked pigs, in 49% of incidents the second case was observed within 1 day after the first case of tail biting in the pen, whereas in 56% of incidents the fourth case was observed within 1 day after the third case of tail biting in the pen (25). The distribution is visualized for the first, second, third, fourth, sixth, and eighth case in Figure 1. Besides the risk of tail biting in the pigs in general, also possible differences due to the genetic potential of the pigs were taken into account.

#### Simulations

The costs of tail biting lesions for different levels of prevalence of lesions were simulated by running the model with different values for parameter α_1_ and then summarizing the results. In addition to the baseline simulation, sensitivity analysis was carried out by running the model with 10% higher price of either pigmeat, piglets, or feed than in the baseline simulation. The benefits of early warning systems were simulated by setting parameter α_2_ or α_3_ equal to zero. Hence, this indicated that the risk of tail biting events after the first incident was not higher than that of the first case.

Prevention and mitigation measures described in section Characterization of Interventions Studied were investigated. The following seven interventions were selected for analysis: (i) straw provision, (ii) improvements in housing ventilation, (iii) improvements in pig genetic pre-disposition to tail bite, (iv) reduced stocking density, (v) provision of point-source enrichment objects, (vi) improvements in herd health, and (vii) early warning systems, including also systems based on precision livestock farming (PLF) technology. These measures were selected for analysis, using the knowledge of experts involved in the GroupHouseNet COST action (https://www.grouphousenet.eu/), on the basis that they were, according to the current scientific knowledge, considered to have the potential to effectively reduce the risk of tail biting lesions and to also be practically implementable on farms. The scientific knowledge on the efficacy of interventions, identified via searching for studies using reference databases, was briefly synthesized to provide quantitative information on the costs of adoption and impacts that the measures have on tail biting incidence. The effects of measures were reviewed in both docked and undocked pig populations.

The measures considered in the modeling were primarily the measures for which information on the costs of adopting the measure has been provided. The ranges of these costs are reported together with modeling results in Table 3. The costs of interventions reported in the next section were compared with the costs of tail biting at different levels of prevalence of tail biting as simulated by the model. The purpose of this comparison was to investigate how large a reduction in tail biting incidence at each initial prevalence level would be required to offset the intervention costs. The model was programmed in, and the simulations were carried out with, MATLAB R2014b 8.4.0.150421 (MathWorks Inc., USA) software.

**Table 3:** The costs (€ per slaughtered pig) of measures to prevent or mitigate tail biting lesions in fattening pigs and minimum change in the tail biting lesions required to cover the lowest or the highest costs of prevention.

**Preventive measure**	**Costs of prevention^a^**		**Required reduction in tail biting lesions to cover the lowest or the highest costs of prevention at different levels (2% through 32%) of initial prevalence^b^**									
			**2%**		**4%**		**8%**		**16%**		**32%**	
	**Max**	**Min**	**Max**	**Min**	**Max**	**Min**	**Max**	**Min**	**Max**	**Min**	**Max**	**Min**
Straw provision	€1.67	€4.17	>100%	>100%	>100%	>100%	93%	>100%	46%	>100%	22%	56 %
			(NA)	(NA)	(NA)	(NA)	(7.4)	(NA)	(8.6)	(NA)	(25.0)	(14.1)
Improvements in ventilation	€0.35	€0.35	72%	72%	38%	38%	19%	19%	10%	10%	5%	5%
			(0.6)	(0.6)	(2.5)	(2.5)	(6.5)	(6.5)	(14.4)	(14.4)	(30.4)	(30.4)
Genetic changes	^c^	€2.70	0%	>100%	0%	>100%	0%	>100%	0%	75%	0%	36%
			(2.0)	(NA)	(4.0)	(NA)	(8.0)	(NA)	(16.0)	(4.0)	(32.0)	(20.5)
Increased space allowance by 0.2 m^b^	^c^	€3.72	0%	>100%	0%	>100%	0%	>100%	0%	>100%	0%	50%
			(2.0)	(NA)	(4.0)	(NA)	(8.0)	(NA)	(16.0)	(NA)	(32.0)	(16.0)
Provision of point-source enrichment objects	€0.03	€1.78	6%	>100%	3%	>100%	2%	99%	1%	49%	0%	24%
			(1.9)	(NA)	(3.9)	(NA)	(7.8)	(0.1)	(15.8)	(8.2)	(32.0)	(24.3)
Improvement in herd health	€1.06	€3.41	>100%	>100%	>100%	>100%	59%	>100%	29%	94%	14%	46%
			(NA)	(NA)	(NA)	(NA)	(3.3)	(NA)	(11.4)	(1.0)	(27.5)	(17.3)
Early warning systems	€0.80	€6.00	>100%	>100%	87%	>100%	45%	>100%	22%	>100%	11%	80%
			(NA)	(NA)	(0.5)	(NA)	(3.6)	(NA)	(12.5)	(NA)	(28.4)	(6.4)

*The numbers in parentheses below each required reduction represent the prevalence after the required reduction has been reached (rounded to nearest decimal). Min represents the smallest cost estimate and max the largest cost estimate according to the literature cited in the Material and Methods section of this study*.

a *€ per slaughtered pig. Estimated based on to the literature cited in the Materials and Methods section*.

b *Initial prevalence levels (2, 4, 8, 16, or 32%) refer to the prevalence of tail biting lesions before applying the selected measure, Interpretation of results: For example, if the initial prevalence of tail lesions is 2% and a 72% reduction is required, then the measure should reduce the prevalence from 2 to 0.56% (2% ×0.72 = 0.56%). If the required reduction is >100%, then the measure was not economically viable in the given situation. NA, Unable to calculate the prevalence after the required reduction has been reached*.

c *Lower estimate for the cost of prevention is not provided because it may be close to negligible in special cases*.

### Characterization of Interventions Studied

#### Straw Provision

Straw provision has been acknowledged as a key measure to mitigate tail biting (16, 27). A review (3), which was mainly based on studies on pigs with intact tails, indicated that light straw (12.5–20 g/pig/day) reduced tail biting on average by about 86% when compared to the control “no enrichment or hanging toy/chain,” or by 72% when compared to “straw rack” as a control. Plentiful straw (at least 500 g/pig/day) reduced tail biting on average by 85–88% when compared to “no enrichment or hanging toy/chain” or by 65% when compared to “straw rack” as a control, while a straw rack reduced tail biting on average by 22% when compared to “no enrichment or hanging toy/chain” as a control. A study (28) in finisher pigs in Denmark indicated that pens with no straw had a 2.22-fold higher risk of tail damage compared with pens with straw provided (150 g/pig/day on the solid floor). Although lowering stocking density from 0.73 to 1.21 m^2^/pig did not affect the risk of tail damage, a combination of the above-mentioned straw provision and lowered stocking density showed a similar risk of tail damage as seen with only tail docking.

The costs of straw provision include the material costs and the additional labor needed to distribute straw and to clean the pens when straw is provided in amounts such that some soiled straw remains in the pen after pigs have explored the enrichment. Straw provision may also require larger slurry pipes so that the liquid manure system is not clogged by straw. Scenarios have been presented (16) where the material and labor costs of daily straw provision were €1.67 or €4.17 per slaughtered pig, for the amount of either 100 or 200 g/day per pig of chopped straw, respectively, when compared to the scenario without any straw.

#### Improvements in Housing Ventilation

Correlating environmental measurements and animal-based indicators from Dutch nurseries and growing–finishing farms showed that tail biting can be one of indicators of suboptimal climatic conditions (29). Inadequate ventilation can be considered as a factor triggering tail biting behavior (30), although quantitative impacts can vary substantially case by case. A farm survey indicated that the use of natural ventilation or mechanically controlled natural ventilation was among the factors that reduced the probability of long-tailed pigs being tail-bitten (31). Having short-tailed pigs has been related to the presence of mechanical ventilation and pens without straw, while having pigs with undocked or long tails has been related to the presence of natural ventilation and straw (32). It has also been reported that an outbreak of tail biting observed during an experiment was probably triggered by a failure of the ventilation system, which resulted in air quality changes such as higher ammonia concentrations and sudden temperature changes (33).

The costs associated with improving ventilation can be 2-fold. On one hand, enhanced ventilation requires investing in appropriate housing and equipment, and this can be costly. However, such investments are typically made when building a new pig house or renovating an existing one. Because a ventilation system is needed in any case, these costs cannot be considered to relate only to tail biting. On the other hand, it is important that the existing ventilation system is monitored and used correctly. Major tail biting outbreaks can occur if there is a failure in ventilation or if it is functioning suboptimally. In this case, regular checks and maintenance service of the ventilation system, rapid response to failures in ventilation, and correct adjustment of the ventilation equipment's parameters are the key to ensure appropriate functioning of the ventilation system. Costs associated with these activities are mainly labor costs, incurred either by farm workers or by service providers taking care of ventilation equipment. For instance, allocating 1 h per week to check the ventilation system functioning, at an hourly labor cost of €16 (23), leads to an annual cost of €832. Information obtained from a ventilation service provider suggests that cleaning and adjustment of the ventilation system once per year costs at least €550. Hence, on a farm producing 4,000 pigs per year, a ventilation check would cost €0.35/pig.

#### Genetics

There is much anecdotal report from pig producers of an association between tail biting prevalence and certain genetic lines of pig. However, despite reports of breed differences in predisposition to be an initiator or recipient of tail biting, published scientific evidence on tail biting associations with genetics is still sparse (6, 10, 19, 34). The only genetic analysis of tail biting behavior published to date (35) showed that predisposition to tail bite had a heritable component within the Landrace population in their study (*h*^2^ = 0.05±0.02, *P* < 0.05 as a 0–1 trait, equivalent to *h*^2^ = 0.27 as a continuous trait), but could not show a measurable heritability in the Large White population, which had a lower overall prevalence of exhibiting the behavior. There may also be a genetic component of similar magnitude to becoming a victim of tail biting. To date, there is no reliable predictive phenotypic characteristic or genetic marker for tail biting predisposition which could be applied in a selection program, although a different expression pattern in 19 genes has been found in neutral pigs compared to performers and receivers of tail-biting behavior (36). In consequence, progress in the direct selection of a low-biting genetic line could currently only be made by utilizing phenotypic data on injurious biting or on tail damage in large populations, although an indirect selection approach using social breeding values for growth rate has shown some promise in reducing tail damage prevalence (37).

The cost of utilizing genetic change to reduce the prevalence of tail biting lesions would come from two sources. The first would be the costs of collecting and analyzing phenotypic data on the trait. The second cost would relate to any reduction in other performance traits which had an unfavorable genetic association with biting behavior, such as lean tissue growth rate (35), i.e., the opportunity cost of advancing low tail biting as a trait instead of other traits. There is no accurate information about the costs of animal breeding programs and which proportion of these costs could be associated with breeding against tail biting. However, some examples exist. For instance, in a French case study overall €2.2–2.7 per produced pig (2% of production cost) was associated with genetic development^1^ One can also postulate scenarios for how large the tradeoffs in other traits might be. For instance, one could test whether the cost of a 5% reduction in daily gain and an increase in carcass fat would be covered by reduced tail biting incidence. While traits with unfavorable genetic associations can be improved in parallel, the genetic improvement in a trait will not be as large as if it would be the only trait included in the breeding evaluation. Hence, the genetic potential for traits such as the daily weight gain and carcass leanness would still improve in parallel with the traits used to measure tail biting, but the improvement would be smaller than when tail biting was not included in a breeding program. Furthermore, it may take several years to achieve the desired genetic progress because animal breeding is a time-consuming process.

#### Stocking Density

Most reviews of the risk factors for tail biting have highlighted stocking density as an important factor (10, 34), although not all studies have found an effect, especially when only relatively high space allowances have been studied (38, 39). A threshold of 110 kg/m^2^, equivalent to 0.9 m^2^ per 100 kg finishing pig, for increased tail biting risk has been suggested (14). However, a higher damage prevalence in finishing farms with stocking density lower or equal to that specified by the EU regulations has been reported (40). While growing pigs (30–40 kg liveweight) housed at <0.31 m^2^ per animal have been reported to exhibit more lesions (41), an approximately linear relationship at both 40 and 90 kg liveweight between space and tail biting damage prevalence over the range 0.7–1.5 m^2^ has also been reported (42). Countries which have banned tail docking generally provide increased space allowance for their finishing pigs, viewing this as an important component of managing tail biting risk [see (43, 44)]. A comparison of pigs housed at 1.21 or 0.73 m^2^, by changing group size in a common pen size, showed only a statistical tendency for a beneficial effect of increased space on the proportion of pens experiencing tail biting, with an overall magnitude of reduction from 57 to 41%. The magnitude of difference was similar in both docked and undocked pigs (28).

The cost of providing more space for finishing pigs relates primarily to the capital cost of housing, increased labor input needed to clean the pens and to other costs associated with the size of housing, such as heating, ventilation, and insurance costs. Whilst increasing space allowance may have some concurrent benefits for growth rate which offset this cost, these might be relatively minor at allowances above the current EU legal requirement (45). Two scenarios (16) involving increased space allowance from 0.7 to 0.9 m^2^ per pig or to 1.0 m^2^ per pig suggested that the additional capital costs associated with these scenarios were €2.52 and 3.78 when 2/3 of the pen area was slatted floor, and the additional labor costs associated with increased space allowance were €1.2–1.8 per pig.

#### Point-Source Enrichment Objects

Especially on farms with fully slatted floors and liquid manure management, it can be challenging to provide pigs with straw, or other bedding-type manipulable material (46). In these cases, point-source objects are often used to provide pigs with some outlet for their intrinsic need to root and chew (47). Such objects are not regarded as optimal, and according to the EU recommendation they should be used in combination with more effective materials, such as straw or other roughage (48). Objects such as fabric sacks (49) and fresh wood (50) have been able to reduce tail biting, and giving multiple objects simultaneously (46, 51) or changing objects regularly (52) also appear to reduce the risk for tail biting damage.

Data collected by the University of Helsinki^2^ showed that 20 cm of fresh wood per pig in the finishing unit cost €1.78 per slaughtered pig. The objects need to be installed in pens and renewed or cleaned when necessary. Hence, the wood was assumed to be renewed twice a year, and the supporting chains every 5 years. The work needed to furnish a pen for 10 pigs, including the cost of acquiring and processing the wood, was estimated based on observations during an experiment to be 1 h 40 min per pen.

Data from Ireland (46) suggested that providing a piece of spruce in the finishing unit cost only about €0.03 per pig when labor costs were not considered. Data collected as part of a Finnish study (51), suggested that adding 10 sisal ropes (a total of 7 m) to farrowing pens incurred a total cost of about €0.51 per piglet, assuming 11 piglets were weaned. Again, this estimate included material and labor costs, and the ropes were renewed for each new litter. A UK-based -guide to environmental enrichment for pigs (53) suggested that the costs of fabric/jute sacks can be at least €0.55 per sack.

#### Improvement in Herd Health

Epidemiological and on-farm studies have indicated a link between reduced health, especially respiratory, enteric and locomotory symptoms, and tail biting (14, 54). For instance, an odds ratio of 3.4 for the risk for locomotory disorders after a pig had been observed to have a bitten tail has been reported. Conversely, the risk of tail biting was elevated after a pig had been observed to have a locomotory disorder, because the odds ratio for tail biting was 1.6 when compared to healthy-legged pig (26). More recently, analysis of registry-based farm data has suggested that tail biting treatment is more likely when there were other health disorders present in the pen, especially joint infections and other locomotory disorders, and vice versa (55). An increased incidence of ear biting and ear damage, but not tail biting in pigs with docked tails, in pigs kept in low sanitary conditions has also been shown (56). Furthermore, there is evidence that vaccination protocols reducing clinical and subclinical disease can reduce the risk for tail biting. Vaccinations against *Lawsonia intracellularis*, Porcine Circovirus Associated Diseases, Porcine Necrotic Ear Syndrome, and Post-weaning Multisystemic Wasting Syndrome have all been linked to a reduction of tail biting lesions in pigs (57–61).

Enhanced hygiene and biosecurity and applying appropriate vaccination protocols were considered as measures to achieve improved herd health. Improvements in ventilation, stocking density and enrichment provision also contribute to herd health and these are considered in other parts of section Characterization of Interventions Studied. It is also relevant to note that other benefits of health interventions will help to offset any costs, as health interventions are not targeted to combat only tail biting. Achieving a better hygiene status requires additional work on a daily basis for activities such as cleaning the pens, especially between batches when rooms can be thoroughly cleaned and even disinfected, and for paying attention to various other practices at the farm. All-in-all-out systems also improve the possibility to maintain good hygiene and pig health, as they allow for proper cleaning between batches, and can lead to economic benefits [e.g., (26)]. It was estimated that cleaning and disinfection takes about 2 min labor input per slaughtered fattening pig (62). Approximately 2 min per pig were estimated also for daily manure handling. At a cost of €16 per h (23), the total labor cost for both of these operations would be €1.06 per pig. Maintaining a high level of biosecurity on farms is essential for the sake of contagious disease risk, and measures such as washing of hands, boots and tools used in the piggery needs to be included in work routines. Costs of vaccination include both the purchase of the substances and supplies for administering the vaccines, as well as increased labor costs for performing the vaccination. These costs can vary by case and disease, e.g., €2.35 per pig against *Actinobacillus Pleuropneumoniae* (21), €1.95 per pig against *Mycoplasma hyopneumoniae* (63), and costs for different diseases ranging up to €1.87 per pig depending on the disease (64).

#### Early Warning Systems, Including PLF-Based Systems

A tail biting outbreak can escalate rapidly once damaged tails are present within the group [e.g., (25, 26)]. The presence of blood stimulates further biting from both the primary biter and other pen-mates (65). For this reason, early detection of a developing problem can facilitate early intervention to prevent escalation and serious damage. The success of intervention depends on the number of damaged pigs at the time it is applied (46). The behavioral changes which occur when an outbreak is imminent have sometimes, but not always, included increased activity, changed feeding and drinking patterns, and increased manipulation of penmates or pen furniture (66). For example, some changes in feeding behavior in pens with tail biting have been observed as early as 9 weeks before an outbreak (67).

The most reliable early warning signal to date is a change in tail posture (68). Studies in weaners and finishing pigs have shown that uncurled and hanging or tucked tails can be seen up to 7 days before a clinical outbreak (69–72). However, the magnitude of change in tail posture may be challenging to detect by daily visual inspections, especially in systems with large pen groups and *ad libitum* feeding (73). As a result, automated PLF systems are now being explored (74). By monitoring pigs in a commercial setting using time-of-flight 3D cameras and machine vision, an algorithm that could detect lowered tail posture of pigs with a good accuracy, and which could be used to warn about a tail biting outbreak a week prior to the event, has been created. This would give pig producers the time to inspect their pigs and take preventive measures to avoid the tail biting outbreak, such as enriching the pens or swapping pigs between pens (74), and would save labor input on observing the pigs and treating pigs with lesions.

The cost of implementing an early warning and intervention system will depend on whether this is done through increased human surveillance or by an automated system. Increased human surveillance will necessitate extra time spent observing animals and thus increase the labor cost associated with pig production. The labor input per pig in the finishing phase varies widely according to the enterprise scale and degree of automation, but is of the order of 0.25 h per finished pig [i.e., about €4 per pig (62, 75)], of which ~0.05 h is attributed to “inspection” and ~0.05 to “veterinary activities” (75). A doubling of the inspection time could therefore increase labor cost per pig in a finishing unit by up to 20%.

An automated system which facilitates an early detection of tail biting events will require capital investment and staff time for erecting and maintaining the camera network. Based on data obtained from a pilot study by Scotland's Rural College, the cost of a 3D camera and fittings is ~€1,250 per pen and the license for software used in multiple pens costs ~€1,000. In the future, a rail system and moving camera might be developed to spread camera costs across more than one pen. With 5–10 years camera lifetime and 6% annual interest rate, the cost per batch would range from €40 to 90. Besides the costs of the early warning system itself, staff time and materials needed to apply intervention measures might include identification and correction of environmental or nutritional deficiencies, moving animals to a new pen, removal of biting or bitten individuals or addition of extra enrichment.

## Results

### Financial Effects of Different Levels of Tail Biting Lesions

**Figure 2:**
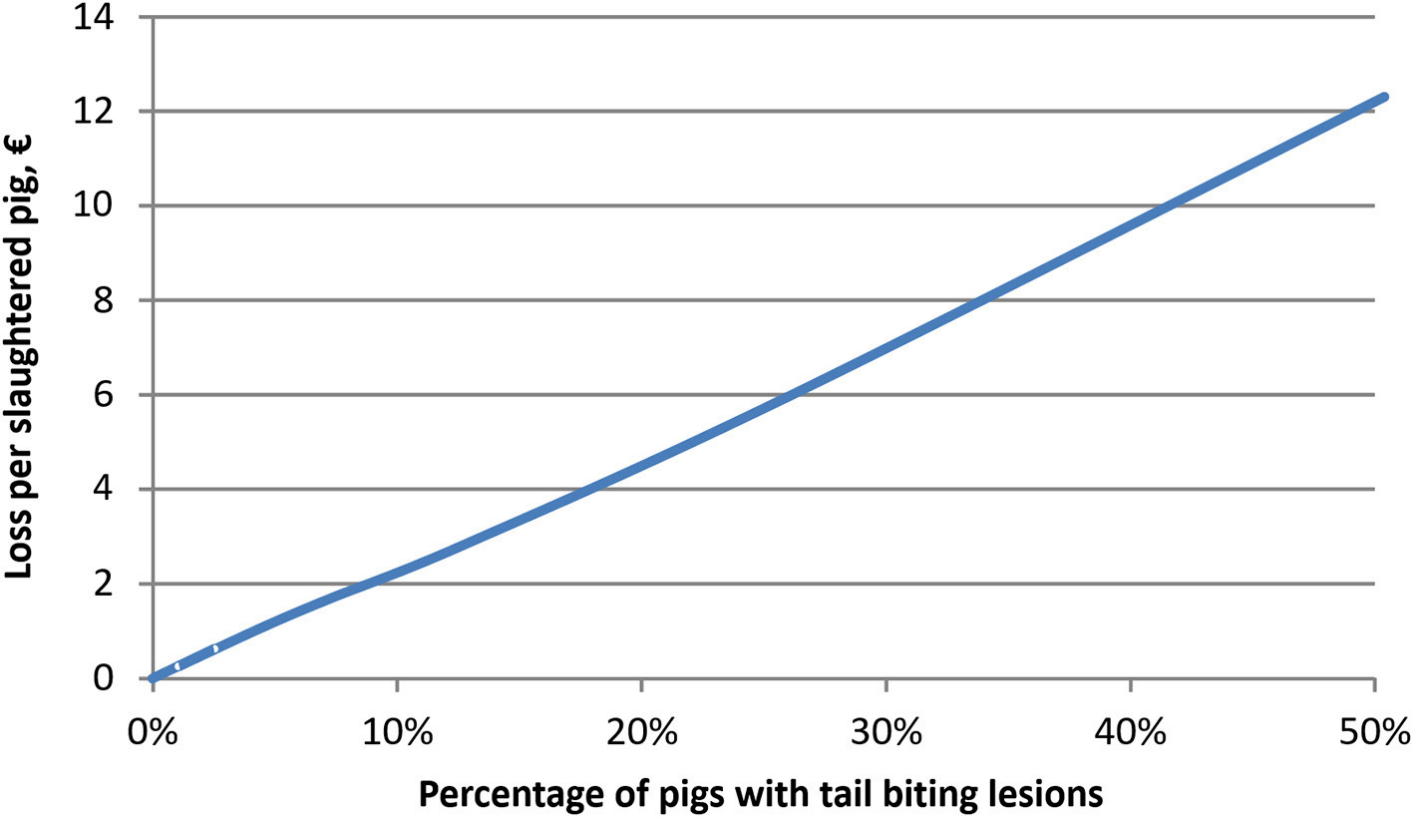
Simulated losses due to tail biting lesions in fattening pigs at different levels of prevalence of lesions (average% pigs in a pen with tail biting lesions).

**Figure 3:**
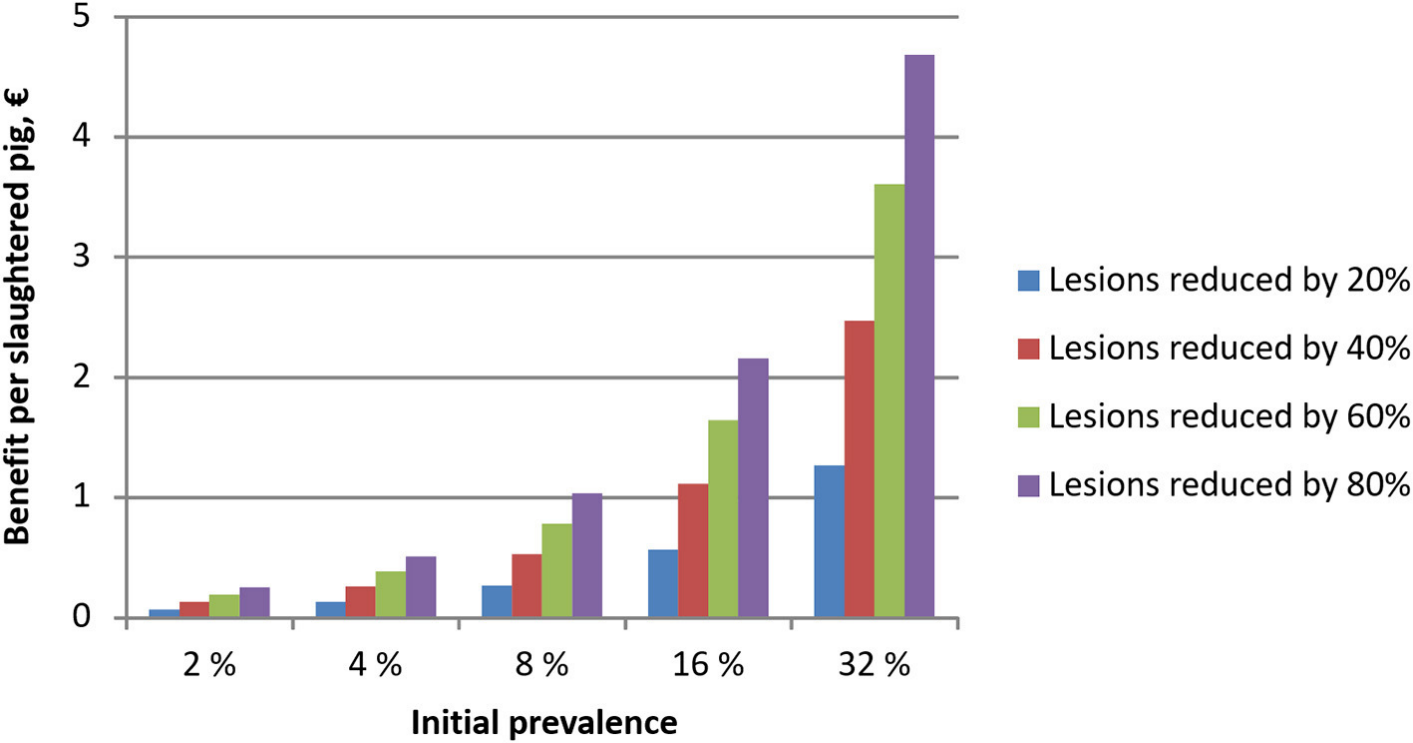
Simulated financial benefits when tail biting lesions in fattening pigs are reduced by 20, 40, 60, or 80% from the initial prevalence level (level before applying an intervention) and the initial prevalence is 2, 4, 8, 16, or 32%. The benefits do not include the cost of an intervention measure.

At farm level, the costs of tail biting lesions were simulated to range up to €2.3 per slaughtered pig [~1.6% of the average carcass value of a pig (with and without lesions)] when the prevalence was increased gradually from zero up to 10%. However, the costs per pig with tail biting lesions were substantially higher, and were simulated to range from €16 to 35 per pig with tail biting lesions, depending on the prevalence of lesions. Figure 2 illustrates how the financial impact of tail lesions per slaughtered pig increased when the prevalence of lesions increased from zero up to 50%. The increase in financial losses was mainly caused by the increasing prevalence of lesions. Figure 3 illustrates the financial benefits per slaughtered pig when the prevalence of tail biting lesions is reduced by 20, 40, 60, or 80% from different initial levels of prevalence. Obviously, the financial benefit from reduced prevalence was the largest when both the initial prevalence and the reduction in the prevalence of tail biting lesions were high.

Table 3 presents estimates for the costs of applying preventive measures according to the literature cited in the Material and Methods section of this paper, as well as the change in tail biting prevalence required to cover the costs of these mitigation measures. The results point out that some of the measures require high initial levels of tail biting lesions before they are cost-effective to be applied, whereas other measures may be beneficial already at low levels of initial prevalence. For instance, measures to mitigate failures in ventilation can be profitable at all levels of initial prevalence whereas straw provision, genetic improvements, increased space allowance and automated early warning systems may require a higher capacity for savings before they are profitable. In general, the more costly prevention measures tended to become gradually financially feasible when the prevalence without applying these preventive measures was 16% or higher.

The challenge in implementing preventive measures profitably at low levels of initial prevalence was the targeting of measures. The better the measures could be targeted to the pigs and pens at greatest risk of experiencing tail biting lesions, the more profitable the measures were. The benefits simulated for an early warning system by setting parameter α_3_ equal to zero (i.e., reducing the risk of tail biting damage after the first case) were <€1 per pig when initial prevalence level was below 10%. However, when parameter α_2_ was also adjusted, the benefits were substantially closer to the numbers reported in Figure 2.

### Sensitivity Analysis

Sensitivity analysis was carried out by increasing the pigmeat, piglet and feed prices by 10% each. When the price of pigmeat was increased by 10% (*ceteris paribus*), the simulated financial benefits associated with reducing tail biting lesions were larger than the benefits reported in Figure 3. At an initial prevalence level of 2–8%, the effects were 10–11% higher, and at an initial prevalence of 16 or 32% the benefits were 8 and 4% higher than those reported for similar baseline scenarios in Figure 3. Moreover, the required reduction in tail biting lesions to cover the costs of prevention at different levels of initial prevalence were 4–11% lower than the estimates reported in Table 3.

A 10% increase in piglet price decreased economic benefits from mitigating tail biting by 2–7% when compared to results presented in Figure 3. Again, the difference between Figure 3 results and sensitivity analysis scenarios were smaller at higher levels of initial prevalence. A 10% increase in feed price, by contrast, only resulted in <2% decrease in economic benefits from mitigating tail biting.

## Discussion

The results suggest that if the prevalence of tail biting lesions was on average 10%, the costs of these tail biting lesions could be up to €2.3 per slaughtered pig. This was ~1.6% of the sale value of an average pig (with or without tail lesions). However, the impact on the profit margin was more substantial, because the profit margin represents only a small fraction of the sale value of a pig. At 10% prevalence of tail lesions, the value function of the baseline simulation was reduced by 17%, and this impact increased with the prevalence of tail lesions. How large a change of prevalence of tail biting lesions was required for a preventive measure to be profitable varied widely according to the investigated measure. Measures which were the least expensive to apply obviously required lower efficacy in reducing tail biting prevalence to become profitable than measures which were the most expensive to adopt. However, literature suggests that the likely efficacy of any intervention also varies by measure. For instance, straw provision has been identified as one of the key measures to prevent tail biting, and it appeared to quickly become profitable once the risk of tail biting lesions in pigs increased up to a high level. In contrast, measures such as improvements in housing ventilation and the provision of point-source enrichment objects were assessed to be less costly and, in the best case, they had the potential to become profitable at low levels of initial prevalence.

In our financial analysis, we did not make any assumptions regarding the efficacy of each measure because it is challenging to obtain accurate data on the effects of preventive measures and on their costs. High between-farm and between-experiment variability exists in the actual incidence of tail biting, the efficacy of preventive measures, and the potential interactions between these measures, implying that parametrizing a bio-economic model by using information from previous literature is challenging. Therefore, we approached the issue from the perspective of looking at how great the efficacy in reducing tail biting would need to be for each measure to be profitable when the costs of implementation would be known. Besides the aforementioned variability, abandoning the policy of tail docking may alter the measures which are seen to be financially attractive (16). In the current analysis, no specific position on whether the farm is practicing tail docking was assumed, because interventions might apply to both docked and undocked pigs if there is a problem. The analysis focused on what difference interventions must make, given that there is a certain initial prevalence of tail biting lesions in the population. The initial level of pigs with damage might be higher in an undocked population (28) and the efficacy of some measures may depend on the docking status.

Based on the presented review, there are several options to cost-effectively reduce the risk of tail biting lesions. Relating incidence reduction achieved by an intervention to the costs of the measure is necessary to make conclusions on cost-effectiveness of measures. Studies carried out in farms or at slaughter lines have reported, in some cases [e.g., (7–9, 19)], levels of prevalence of wounds which are high enough to justify the more costly measures, while in other cases [e.g., (9, 10)] the reported prevalences have been below a level which justifies the more costly measures considered in Table 3. However, in the latter case the prevalence levels often relate to docked pigs.

The results suggest that improvements in ventilation, genetic change or provision of point-source enrichment objects can be financially viable, even when either the prevalence of tail biting lesions before implementing a measure or the efficacy of the measure is low, because these measures can be quite inexpensive to adopt. A previous publication (16) also emphasized the role of keeping the implementation costs of measures low. The time scales involved in implementing different measures may vary. Whilst provision of point-source objects or ventilation maintenance can be implemented very quickly, other measures involving changes in genetics or housing infrastructure require longer term planning but are likely to have greater overall effect. Whilst it takes a long time for breeding companies to select appropriate genotypes with lower tail biting pre-disposition, once they are available a farm can achieve responses quite quickly, either by buying different stock for fattening, or by using different semen to generate offspring. The results suggest that for farms struggling with more severe outbreaks of tail biting lesions, straw provision, improvement in herd health, increased space allowance by 0.2 m^2^ or investment into an early warning system can all be profitable if their costs can be kept at a reasonable level and the measures resolve the problem that is causing elevated risk of tail biting lesions.

Straw is an effective manipulable material [e.g., (16, 28)] and used commonly in some countries, but it can also be a challenging material to handle and may imply an increase in labor cost. For instance, in a survey, the majority (87%) of Finnish pig producers used straw and newspapers as manipulable materials to reduce the risk of tail biting (43) but, as noted earlier (76), straw provision can increase blockage on the slatted floor. This type of material, if not removed frequently, can result in the clogging of the manure drainage system, which eventually may lead to poorer pen cleanliness and incur additional labor cost to keep the pens clean. In addition, the costs of administering materials such as straw must be low enough because the measure is repeated often, so that the cost accumulated over the pig's lifetime can be substantial.

The qualities of intervention measures also matter, and the impact of measures may vary case by case. At an early stage, straw may be effective in reducing nosing and tail biting (77), and access to straw in the pre-weaning period may reduce mounting and oral manipulation directed at other individuals (78). Straw elicits active, diverse behaviors in pigs and reduces manipulatory social behaviors such as tail biting. However, reducing the length of straw, by chopping it for easier handling, diminishes these positive effects (79). In a Dutch study, the provision of a small amount of long straw (10 g/pig) twice per day considerably reduced the occurrence of bite marks and tail wounds in weaned piglets compared to the provision of a chain or rubber hose, while a straw rack showed an intermediate effect (80). A Swedish study found that finishing pigs that received more straw had less tail damage, while the effect was less evident in grower pigs (76).

Studies have shown that non-straw enrichment can also effectively reduce tail biting (81). However, it is important to use objects which are of relevance for the pigs, i.e., those which increase species-specific behavior (50), and regrettably, many of the objects (such as chains and dry wood blocks) used commonly on farms have not been proven to be very effective (81). Objects such as fabric sacks (49) and fresh wood (50) have been able to reduce tail biting, and giving multiple objects simultaneously (46, 51) or changing objects regularly (52) also appears to reduce the risk for tail biting damage. Objects have also been successfully used to intervene when a tail biting outbreak is already ongoing (43, 82), and it has been suggested by pig producers that it is important to make sure that novel objects are available for use in case of outbreaks (43). However, it is not yet certain what degree of provision of point-source objects is adequate, and a low number or amount might even increase tail biting due to increased competition over the object (46).

Genetic selection for reduced susceptibility of tail biting behavior and for tail biting lesions also represents a relevant measure. Although it may take several years to achieve the desired genetic progress, once achieved, the result can be widely available. The cost of collecting and analyzing phenotypic data on the trait is likely to be reduced in the future by the growing use of PLF approaches in both farms and abattoirs. The extent of any loss of progress in other performance traits which have an unfavorable genetic association with biting behavior is still uncertain. Within the Landrace breed, tail biting predisposition has been shown to have a positive genetic correlation with lean tissue growth rate and a negative genetic correlation with subcutaneous fat thickness at the P_2_ position, indicative of carcass fat content (35). In line with this, gilts with high levels of tail or ear biting have been seen to have a higher genetic potential for several production traits (49). This implies that selection for reduced tail biting predisposition might involve a reduction in growth rate, carcass leanness and feed efficiency traits. However, pigs selected for greater feed efficiency seem to be less affected by tail biting, possibly because of a lower level of physical activity (83). Quantification of effects is challenging, as data comparing the performance of tail biting, victim and control animals generally involve confounding of cause and effect, and therefore more detailed analyses of genetic interventions are recommended.

As noted earlier, epidemiological and on-farm studies have indicated a link between reduced health, especially respiratory, enteric and locomotory symptoms, and tail biting. The underlying mechanism whereby decreased health might be a risk factor for tail biting lesions is uncertain, but suggestions have included sickness-induced changes in cytokines and neurotransmission (84, 85) or involvement of the brain-gut-microbiota axis (86, 87). Colostrum intake, postnatal environmental hygiene, and antimicrobial treatments all affect the development of gut microbiota, immunity and subsequent health, and can thus be important for behavioral development (54, 86). Further research on the interactions of pig health in general and tail biting would be warranted, including the synergies of overall health management in reducing the costs of tail biting and other disorders.

Early detection of a developing tail biting problem can facilitate early intervention to prevent escalation and serious damage. The success of an intervention depends on the number of damaged pigs at the time that it is applied (46). Already explored automated PLF systems using time-of-flight 3D cameras and machine vision can detect lowered tail posture of pigs with a good accuracy and warn about the risk of a tail biting outbreak a week prior to the event (74). This would give pig producers the time to inspect their pigs and take preventive measures to avoid the tail biting outbreak, such as enriching the pens or relocating pigs. However, use of an automated system may also entail additional time in checking out “false positives” which are currently flagged up by the system. The costs of such systems appear still to be quite high for commercial use, but further development can be expected to make the technology more competitive. The cost effectiveness of PLF systems can also be improved by developments where the same devices and systems can be used to simultaneously detect multiple health disorders and to enhance the control of production, such as feeding, growth monitoring and slaughter timing.

One of the challenges concerning the profitability of preventive measures at low levels of initial tail biting prevalence is the targeting of measures, which is related also to early detection of an emerging problem. The better the measures can be targeted to the pigs and pens at risk of tail biting lesions, the more profitable the measures can be. The application of Decision Support Tools focused on the individual farm situation can therefore be a key part of any strategy [e.g., (86, 88)]. Because of the multifactorial nature of the tail biting problem, straw-provision, or any other intervention, may not be able to adequately control tail biting on its own if other risk factors are ignored. For example, in high stocking density conditions, the prevalence of tail lesions can be high even when the animals have access to straw racks and, in such cases, increasing the space allowance can be an important factor to reduce tail biting (89). The genetic background of pigs can also contribute to tail biting occurrence regardless of the enrichment provided (90). In our analysis, we have looked at each intervention separately. Although resolving the problem of tail biting may require making multiple interventions simultaneously, the current scientific literature does not provide robust data to parametrise how interactions between the interventions impact their aggregate efficacy. It cannot be concluded that they are additive, since if tail biting is abolished by one measure, then another may have no possibility for effect. Hence, further research on these interactions would be needed. Furthermore, the interventions may result in other interactive benefits. Increased space allowance, for instance, not only directly reduces tail biting (10, 34), but it can also provide other health benefits such as reduced incidence of respiratory disorders. Therefore, the costs of secondary infections were factored in the parameters of the current model.

The decreasing difference between the baseline and sensitivity analysis scenarios as the tail biting prevalence increases, shows how the cost effectiveness of intervention measures may vary as other production circumstances change. The result that obtainable benefits were larger at higher pigmeat price and smaller at higher feed or piglet prices was related to the fact that the economic return of pig farming increases when pigmeat price is raised and decreases when input prices are raised. Hence, at higher (lower) level of economic return the producer has more (less) to lose, should a tail biting outbreak occur. While fixed costs per slaughtered pig are increased when a tail biting outbreak occurs, at the farm level a tail biting outbreak does not change the fixed costs. By contrast, both variable costs and economic return on pig fattening are changed both at the farm-level and per slaughtered pig, when an outbreak occurs. The modeling approach used in this study takes these aspects into account.

Tail biting lesions are both an animal welfare and animal health issue. The principal results are applicable also in the broader context of animal health and welfare issues. Interventions tend to become economically more viable when the risk of a health disorder is increased, the efficacy of an intervention is improved or the cost of adopting an intervention is decreased, as has been observed in the animal disease literature. Increased profitability of production may imply that also the benefits of improved control of health and welfare issues are greater than they would be at a lower level of profitability (e.g., with a lower meat price). However, this does not always lead to enhanced control of disease, because other management decisions may be substitutes for enhanced disease control and the benefits of substitute measures also influence which are the most profitable measures. For example, if pigs with poor health are lagging behind the growth of other pigs, the farmer may mitigate disease losses by both improving the health of pigs and slaughtering pigs with poor health at a lower weight.

Previous research on economic aspects of tail biting in pigs is scarce and earlier estimates are often based on either expert knowledge (14) or cost calculations [e.g., (15)]. While two recent studies have used a stochastic decision tree model (16) and a stochastic budgetary simulation with bio-economic model (17), the authors are not aware of peer-reviewed studies which have integrated the dynamics of tail biting in the pen and profit maximization when analyzing the economics of tail biting. The dynamic approach which maximizes the return on a pig space unit allows the analysis of management issues in greater detail. For example, the role of early warning signals, the timing of interventions and the role of information in a farmer's decision making could be further analyzed with the present model. Modeling the dynamics of the pig production process with a disease condition requires quite detailed data to parametrize the model's equations. Our background data suggest that the spread of tail biting lesions can be modeled as a dynamic process which has similarities with modeling the spread of pathogens. However, one of the benefits of our approach is that, when parametrized appropriately, it can take into account that farm management decisions can change case by case and that this can the impact on both the benefits of interventions and the impacts of tail biting lesions. A similar dynamic modeling approach can also be applied more broadly to study the economics of controlling production diseases and infectious diseases in a livestock farm.

While the farmer as a decision-maker was considered risk neutral, the model itself can take into account risk aversion over time. This is because of the afore-mentioned dynamics and recursive structure of the model's equations. Moreover, it can be further developed toward an application of dynamic programming and real options which utilize these structures. Another direction for further research could be to apply linear programming methods such as MOTAD [Minimization of Total Absolute Deviation; e.g., Target-MOTAD (91)], which aims to minimize the deviations of the maximized value and hence pay attention to both the expected profit and the variation of this. In economics, risk is often considered through expected value and variance. While the current model maximized the expected value of the value function, applying MOTAD or an expected value-variance approach in the current model would also add the variation of maximized value from its mean as a cost to the revenue function.

The current study provides insights on the conditions under which an intervention to reduce the risk of tail biting lesions can be economically viable. Economic analysis of pig producer decision-making focuses on how scarce resources can be allocated to obtain the best outcome. It therefore requires identifying different implications of decisions made by the pig producer. Besides the productivity gains achieved because of reduced tail biting lesions, there can be other benefits associated with interventions. These include other health benefits than those associated with tail biting and secondary infections, and a price premium which may be obtainable from the market when an intervention can be used to deliver perceived higher-value products to the consumers, which was not considered in this study. In addition, pig producers' incentives to implement an intervention may be different from the incentives faced by other stakeholders, experts or society [see Ref. (92) for discussion]. The perceived importance of risk factors (and preferences to control the risk of tail biting) may differ between scientists and pig producers, and between pig producers in different cultures of pig production (43). This indicates that a scientist-farmer dialogue, as well as international communication, is important when trying to reduce the risk of tail biting and the need for tail docking. Moreover, this study suggests that there is uncertainty about the costs and benefits related to the pig producers' decision making and therefore further research on pig producers' financial and non-financial incentives to mitigate the risk of tail biting lesions in different contexts is needed.

## Conclusion

The results provide guidance on the expected benefits and costs of several studied interventions. The spread of tail biting lesions and the economics of controlling the risk of lesions at the farm level can be modeled as a dynamic process, using an approach which can also be used to model the spread and control of pathogens. The change in prevalence of tail biting lesions required for a preventive measure to be profitable varies according to the costs of the measure and the initial prevalence of tail biting, not just the efficacy of the measure to reduce tail biting lesions. Measures which were considered the least expensive to apply, such as maintaining good housing ventilation, were observed to become profitable at a lower level of efficacy than measures which were the most expensive to apply. Measures which were more efficient in reducing tail biting lesions but also more expensive to implement, such as straw provision, can be considered as cost-effective when the risk of tail biting is high. At lower risk levels, the provision of point-source enrichment objects and other less costly but relatively less effective measures can play an important role. However, appropriate targeting of the measures is essential for their profitability because an intervention is not automatically always effective, and the selection of an intervention must be problem-oriented. The preventive measures must therefore be selected for each farm on the basis of both their efficacy and relevance.

More generally, measures affecting animal health and welfare tend to become economically more viable when the risk of a health disorder is increased, the efficacy of a measure is improved or the cost of adopting a measure is decreased. Increased profitability of production tends to imply that the benefits of improved control of health and welfare issues are also greater than they would be at lower level of profitability. Further research could extend the analysis by considering risk in terms of expected value-variance and taking into account a farmer's risk preferences, especially risk aversion which can influence the incentives to apply risk mitigation measures, and by considering interactions between simultaneously applied measures.

## Data Availability Statement

The data supporting the conclusions of this article are available from the corresponding author upon request.

## Author Contributions

JN, AV, and SE conceived the study and study concept. JN, AV, SE, and AW contributed to the model development. All authors contributed to the concept of the study, literature review, discussion, conclusions, and to the writing of the article.

## Funding

This study was produced as part of the COST action CA15134 GroupHouseNet (Synergy for preventing damaging behavior in group housed pigs and chickens). COST is supported by the EU Framework Programme Horizon 2020. JN has received funding from Oiva Kuusisto foundation.

## Conflict of Interest

The authors declare that the research was conducted in the absence of any commercial or financial relationships that could be construed as a potential conflict of interest.

## Publisher's Note

All claims expressed in this article are solely those of the authors and do not necessarily represent those of their affiliated organizations, or those of the publisher, the editors and the reviewers. Any product that may be evaluated in this article, or claim that may be made by its manufacturer, is not guaranteed or endorsed by the publisher.

## Footnotes

^1^ https://www.nucleus-sa.com/media/leaflet_nucleus_forum__004156800_1040_11042013.pdf

^2^ https://slideplayer.fi/slide/2939984/
